# (Benzoato-κ*O*)chlorido[(–)-sparteine-κ^2^ *N*,*N*′]zinc(II)

**DOI:** 10.1107/S1600536809033625

**Published:** 2009-08-29

**Authors:** José Luis Alcántara-Flores, Nayeli Arias-López, Sylvain Bernès, René Gutiérrez, Yasmi Reyes Ortega

**Affiliations:** a Centro de Química, Instituto de Ciencias, Universidad Autónoma de Puebla, Edif. 103F Complejo de Ciencias CU, San Manuel, 72570 Puebla, Pue., Mexico; b DEP Facultad de Ciencias Químicas, UANL, Guerrero y Progreso S/N, Col. Treviño, 64570 Monterrey, NL, Mexico; c Laboratorio de Síntesis de Complejos, Facultad de Ciencias Químicas, BUAP, AP 1067, 72001 Puebla, Pue., Mexico

## Abstract

The title complex, [Zn(C_7_H_5_O_2_)Cl(C_15_H_26_N_2_)], used for the magnetic dilution of the analogous Cu^II^ complex, was synthesized through a direct synthesis route. The coordination geometry around Zn^II^ is best described as distorted tetra­hedral, the largest deviation arising from the (–)-sparteine ligand, as is invariably found in complexes containing this rather rigid mol­ecule. The benzoate anion behaves as a monodentate ligand, with a non-coordinating Zn⋯O separation of 2.969 (5) Å. Mol­ecules are packed in the crystal without significant inter­molecular inter­actions. The shortest Zn⋯Zn separation [6.8186 (7) Å] is observed between mol­ecules related through the 2_1_ screw axis. This is an important feature for the magnetic behaviour of the Cu^II^ analogue, which is intended for modeling isolated metal centers in the active site of type 1 blue copper proteins.

## Related literature

For related Zn^II^ and Cu^II^ complexes bearing sparteine as ligand, see: Alcántara-Flores, Bernès *et al.* (2003 ▶); Alcántara-Flores, Vázquez-Bravo *et al.* (2003 ▶); Jasiewicz *et al.* (2005 ▶); Lee *et al.* (2002 ▶): Reyes-Ortega *et al.* (2006 ▶). For the *κO*-coord­ination mode of benzoate, see: Shanmuga Sundara Raj *et al.* (2000 ▶).
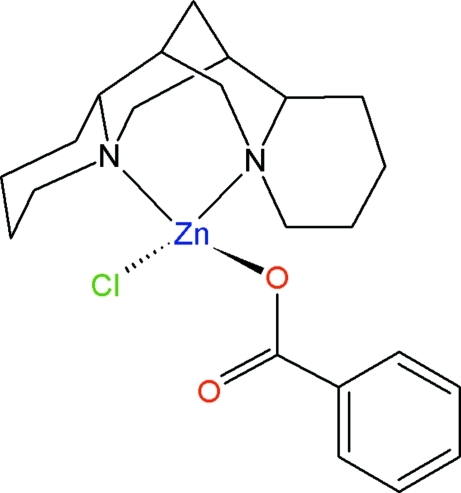


## Experimental

### 

#### Crystal data

[Zn(C_7_H_5_O_2_)Cl(C_15_H_26_N_2_)]
*M* *_r_* = 456.31
Monoclinic, 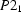

*a* = 8.7784 (9) Å
*b* = 11.8238 (13) Å
*c* = 10.8438 (11) Å
β = 109.671 (8)°
*V* = 1059.84 (19) Å^3^
*Z* = 2
Mo *K*α radiation
μ = 1.31 mm^−1^
*T* = 296 K
0.34 × 0.26 × 0.04 mm

#### Data collection

Bruker P4 diffractometer
Absorption correction: ψ scan (*XSCANS*; Siemens, 1996 ▶) *T*_min_ = 0.760, *T*_max_ = 0.951
3583 measured reflections
2166 independent reflections
1812 reflections with *I* > 2σ(*I*)
*R*_int_ = 0.033
2 standard reflections every 48 reflections intensity decay: 1.5%

#### Refinement

*R*[*F* ^2^ > 2σ(*F* ^2^)] = 0.033
*wR*(*F* ^2^) = 0.069
*S* = 1.01
2166 reflections
254 parameters
1 restraint
H-atom parameters constrained
Δρ_max_ = 0.36 e Å^−3^
Δρ_min_ = −0.31 e Å^−3^
Absolute structure: Flack (1983 ▶), 199 Friedel pairs
Flack parameter: −0.001 (16)

### 

Data collection: *XSCANS* (Siemens, 1996 ▶); cell refinement: *XSCANS*; data reduction: *XSCANS*; program(s) used to solve structure: *SHELXS97* (Sheldrick, 2008 ▶); program(s) used to refine structure: *SHELXL97* (Sheldrick, 2008 ▶); molecular graphics: *Mercury* (Macrae *et al.*, 2006 ▶); software used to prepare material for publication: *SHELXL97*.

## Supplementary Material

Crystal structure: contains datablocks I, global. DOI: 10.1107/S1600536809033625/kj2131sup1.cif

Structure factors: contains datablocks I. DOI: 10.1107/S1600536809033625/kj2131Isup2.hkl

Additional supplementary materials:  crystallographic information; 3D view; checkCIF report

## Figures and Tables

**Table:** 

Zn1—O2	1.940 (3)
Zn1—N1	2.077 (4)
Zn1—N16	2.101 (4)
Zn1—Cl1	2.2189 (13)

**Table:** 

O2—Zn1—N1	114.94 (15)
O2—Zn1—N16	98.37 (14)
N1—Zn1—N16	89.06 (14)
O2—Zn1—Cl1	113.80 (12)
N1—Zn1—Cl1	124.97 (12)
N16—Zn1—Cl1	107.58 (10)

**Fig. 1.**
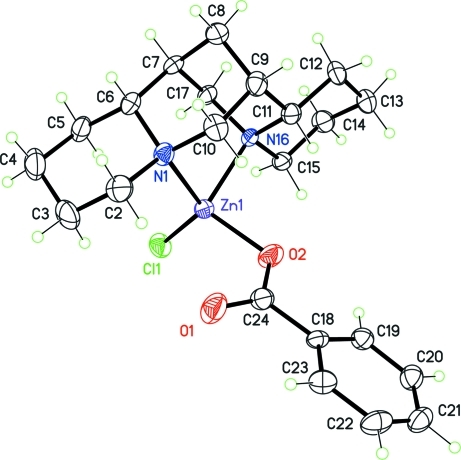
The title molecule with displacement ellipsoids for non-H atoms shown at the 30% probability level. Hydrogen atoms are shown as spheres of arbitrary radius.

**Fig. 2.**
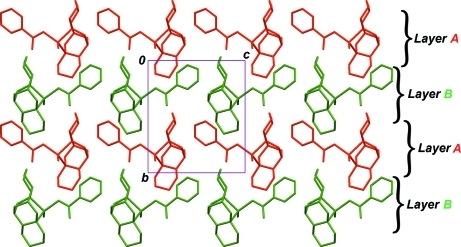
A part of the crystal structure of the title compound, viewed down [100]. The color scheme is used for the sake of clarity and H atoms have been omitted.

## supplementary crystallographic information

### Comment

The search for suitable structural, spectroscopic and magnetic models for type 1 blue copper proteins remains an active field. The title complex deals with such researches; it was first obtained as a by-product during the magnetic dilution of the analogous Cu^II^ complex. Unfortunately, although we accumulated a number of spectroscopic and magnetic data for the Cu^II^ complex, we were unable to get suitable single crystals for its accurate X-ray structural characterization. The structure of the title Zn^II^ complex is, however, a first approach for solving this problem.

The title Zn^II^ complex crystallizes in a chiral space group with the molecule placed in a general position. The (–)-sparteine ligand has the expected *RSSS* absolute configuration and coordinates to the Zn^II^ ion trough N atoms. A benzoate ligand is *κO*-bonded by a single O atom, a mode of coordination documented for Zn^II^ complexes, albeit not very common (Shanmuga Sundara Raj *et al.*, 2000). The non-bonding Zn···O intramolecular separation, 2.969 (5) Å, makes clear that (I) is a four-coordinated complex. The last coordination site is occupied by a Cl^-^ ion, at the expected distance. The local geometry around Zn is better described as tetrahedral distorted (Fig. 1), the largest deviation arising from the (–)-sparteine ligand, as invariably found in complexes containing this rather rigid molecule. The dihedral angle between N1/Zn1/N16 and Cl1/Zn1/O2 planes is 87.25 (12)°, reflecting the steric hindrance of the sparteine ligand.

The title complex is closely related to paramagnetic Cu^II^ complexes [Cu((–)sparteine)(PhCOO)*X*], for which we reported X-ray structures (*X* = Cl: Alcántara-Flores, Vázquez-Bravo *et al.*, 2003; *X* = Br: Reyes-Ortega *et al.*, 2006). However, Cu^II^ complexes are clearly five-coordinated species, with the benzoate behaving as a bidentate κ^2^*O*,*O*'-ligand. This difference is consistent with an atomic radius larger for Cu^II^ than for Zn^II^, and with a further electron withdrawing capacity for the *d*^9^ metal ion compared to *d*^10^ ions. Finally, it should be mentioned that with Zn^II^ as metal center, sparteine-containing complexes more symmetrical than the title complex have been obtained, by coordinating two identical carboxylato-*κ*^1^*O* ligands or by using α-isosparteine (*e.g*. Jasiewicz *et al.*, 2005).

In the crystal structure, the molecules pack at van der Waals distances into two different alternating layers (*A* and *B*, see Fig. 2) parallel to the plane (010). All molecules in a layer have the same spatial orientation. Neighboring *A* and *B* layers are related by a twofold screw 2_1_ axis. An important criterion for the use of these molecules as models for the active site in type 1 copper proteins is the metal-metal separation, which should be as long as possible, in order to mimic magnetically isolated Cu^II^ centers in the native proteins. In the case of the title complex, this distance is 6.8186 (7) Å, and is thus intermediate between separations found in dimorphic dibromo-[(–)-sparteine]-zinc(II) complex, for which metal separations were observed at 6.534 Å (orthorhombic polymorph: Lee *et al.*, 2002) or 7.4715 (6) Å (triclinic polymorph: Alcántara-Flores, Bernès *et al.*, 2003).

### Experimental

Equimolar amounts (1.5 mmol) of zinc powder, (–)-sparteine, benzoyl chloride and DMSO (4.5 ml) were placed in a flask and the mixture was kept under magnetic stirring at 338 K for 8 h. The reaction mixture was then filtered and allowed to stand for 26 days at room temperature, after which a solid material, identified as the title complex, was filtered off (25% yield) and recrystallized from methanol. *M*.p. 459–461 K. A complete spectroscopic characterization was carried out, which is in agreement with the X-ray structure (see archived CIF).

### Refinement

All H atoms were placed in idealized positions and refined as riding to their carrier C atoms, with bond lengths fixed to 0.93 (aromatic CH), 0.97 (methylene CH_2_), and 0.98 Å (methine CH). Isotropic displacement parameters were calculated as *U*_iso_(H) = 1.2*U*_eq_(carrier atom). The absolute configuration was assigned by refinement of a Flack parameter, and agrees the chirality expected from the synthetic route.

### Crystal data

**Table:**

[Zn(C_7_H_5_O_2_)Cl(C_15_H_26_N_2_)]	*F*(000) = 480
*M**_r_* = 456.31	*D*_x_ = 1.430 Mg m^−^^3^
Monoclinic, *P*2_1_	Melting point = 459–461 K
Hall symbol: P 2yb	Mo *K*α radiation, λ = 0.71073 Å
*a* = 8.7784 (9) Å	Cell parameters from 49 reflections
*b* = 11.8238 (13) Å	θ = 3.7–10.8°
*c* = 10.8438 (11) Å	µ = 1.31 mm^−^^1^
β = 109.671 (8)°	*T* = 296 K
*V* = 1059.84 (19) Å^3^	Plate, colourless
*Z* = 2	0.34 × 0.26 × 0.04 mm

### Data collection

**Table:**

Bruker P4 diffractometer	1812 reflections with *I* > 2σ(*I*)
Radiation source: fine-focus sealed tube	*R*_int_ = 0.033
graphite	θ_max_ = 25.0°, θ_min_ = 2.0°
ω scans	*h* = −10→5
Absorption correction: ψ scan (*XSCANS*; Siemens, 1996)	*k* = −14→1
*T*_min_ = 0.760, *T*_max_ = 0.951	*l* = −12→12
3583 measured reflections	2 standard reflections every 48 reflections
2166 independent reflections	intensity decay: 1.5%

### Refinement

**Table:**

Refinement on *F*^2^	Hydrogen site location: inferred from neighbouring sites
Least-squares matrix: full	H-atom parameters constrained
*R*[*F*^2^ > 2σ(*F*^2^)] = 0.033	*w* = 1/[σ^2^(*F*_o_^2^) + (0.0305*P*)^2^] where *P* = (*F*_o_^2^ + 2*F*_c_^2^)/3
*wR*(*F*^2^) = 0.069	(Δ/σ)_max_ < 0.001
*S* = 1.00	Δρ_max_ = 0.36 e Å^−^^3^
2166 reflections	Δρ_min_ = −0.30 e Å^−^^3^
254 parameters	Extinction correction: *SHELXL97* (Sheldrick, 2008), Fc^*^=kFc[1+0.001xFc^2^λ^3^/sin(2θ)]^-1/4^
1 restraint	Extinction coefficient: 0.0038 (9)
0 constraints	Absolute structure: Flack (1983), 199 Friedel pairs
Primary atom site location: structure-invariant direct methods	Flack parameter: −0.001 (16)
Secondary atom site location: difference Fourier map	

### Fractional atomic coordinates and isotropic or equivalent isotropic displacement parameters (Å^2^)

**Table:**

	*x*	*y*	*z*	*U*_iso_*/*U*_eq_	
Zn1	0.35428 (6)	0.83945 (5)	0.07075 (4)	0.03626 (17)	
Cl1	0.59957 (15)	0.88938 (12)	0.07478 (13)	0.0568 (4)	
O1	0.1273 (6)	0.8988 (4)	−0.1933 (5)	0.0736 (14)	
O2	0.2371 (4)	0.7472 (3)	−0.0791 (3)	0.0593 (10)	
N1	0.2098 (5)	0.9334 (3)	0.1500 (4)	0.0395 (10)	
C2	0.1470 (6)	1.0382 (5)	0.0733 (5)	0.0561 (15)	
H2B	0.0728	1.0757	0.1090	0.067*	
H2C	0.0874	1.0180	−0.0168	0.067*	
C3	0.2813 (8)	1.1188 (5)	0.0761 (7)	0.0694 (18)	
H3B	0.2353	1.1865	0.0271	0.083*	
H3C	0.3511	1.0836	0.0343	0.083*	
C4	0.3824 (8)	1.1517 (5)	0.2180 (7)	0.0728 (19)	
H4B	0.4751	1.1964	0.2183	0.087*	
H4C	0.3173	1.1967	0.2562	0.087*	
C5	0.4390 (6)	1.0444 (5)	0.2981 (6)	0.0540 (14)	
H5A	0.5153	1.0051	0.2661	0.065*	
H5B	0.4949	1.0648	0.3888	0.065*	
C6	0.2997 (6)	0.9655 (4)	0.2909 (5)	0.0439 (12)	
H6C	0.2239	1.0088	0.3212	0.053*	
C7	0.3460 (5)	0.8603 (4)	0.3779 (4)	0.0414 (14)	
H7B	0.4003	0.8863	0.4679	0.050*	
C8	0.1909 (6)	0.8004 (5)	0.3750 (5)	0.0534 (14)	
H8C	0.2153	0.7382	0.4369	0.064*	
H8D	0.1193	0.8527	0.3977	0.064*	
C9	0.1123 (6)	0.7570 (4)	0.2361 (5)	0.0448 (12)	
H9A	0.0111	0.7195	0.2320	0.054*	
C10	0.0684 (5)	0.8584 (5)	0.1438 (5)	0.0473 (15)	
H10A	0.0196	0.8311	0.0548	0.057*	
H10B	−0.0121	0.9034	0.1647	0.057*	
C11	0.2198 (5)	0.6685 (4)	0.2039 (5)	0.0396 (12)	
H11A	0.1685	0.6491	0.1112	0.047*	
C12	0.2309 (6)	0.5589 (5)	0.2815 (5)	0.0481 (13)	
H12A	0.2717	0.5761	0.3744	0.058*	
H12B	0.1233	0.5272	0.2614	0.058*	
C13	0.3404 (7)	0.4711 (5)	0.2515 (6)	0.0583 (15)	
H13A	0.3503	0.4058	0.3078	0.070*	
H13B	0.2936	0.4463	0.1613	0.070*	
C14	0.5068 (7)	0.5220 (5)	0.2737 (5)	0.0523 (14)	
H14A	0.5744	0.4677	0.2492	0.063*	
H14B	0.5580	0.5399	0.3658	0.063*	
C15	0.4904 (6)	0.6294 (4)	0.1917 (4)	0.0389 (11)	
H15A	0.5971	0.6616	0.2086	0.047*	
H15B	0.4481	0.6090	0.0997	0.047*	
N16	0.3824 (4)	0.7178 (3)	0.2180 (3)	0.0312 (8)	
C17	0.4586 (5)	0.7755 (4)	0.3460 (4)	0.0400 (11)	
H17A	0.5551	0.8147	0.3447	0.048*	
H17B	0.4915	0.7190	0.4148	0.048*	
C18	0.0832 (5)	0.7189 (4)	−0.2985 (4)	0.0352 (11)	
C19	0.1471 (6)	0.6117 (4)	−0.2996 (5)	0.0427 (12)	
H19A	0.2313	0.5866	−0.2269	0.051*	
C20	0.0874 (6)	0.5418 (5)	−0.4072 (5)	0.0517 (14)	
H20A	0.1322	0.4705	−0.4067	0.062*	
C21	−0.0384 (6)	0.5774 (6)	−0.5152 (6)	0.0551 (16)	
H21C	−0.0783	0.5305	−0.5878	0.066*	
C22	−0.1042 (6)	0.6824 (6)	−0.5147 (5)	0.0584 (16)	
H22C	−0.1906	0.7056	−0.5871	0.070*	
C23	−0.0445 (5)	0.7555 (5)	−0.4080 (4)	0.0475 (13)	
H23C	−0.0888	0.8271	−0.4097	0.057*	
C24	0.1522 (7)	0.7978 (6)	−0.1842 (6)	0.0446 (14)	

### Atomic displacement parameters (Å^2^)

**Table:**

	*U*^11^	*U*^22^	*U*^33^	*U*^12^	*U*^13^	*U*^23^
Zn1	0.0429 (3)	0.0340 (3)	0.0326 (2)	0.0013 (3)	0.01358 (19)	0.0000 (3)
Cl1	0.0589 (8)	0.0505 (7)	0.0747 (8)	−0.0089 (7)	0.0404 (7)	−0.0045 (7)
O1	0.100 (4)	0.049 (3)	0.059 (3)	0.014 (3)	0.010 (2)	−0.010 (2)
O2	0.078 (2)	0.055 (2)	0.0324 (18)	0.005 (2)	0.0018 (17)	−0.0018 (18)
N1	0.044 (2)	0.032 (2)	0.041 (2)	0.006 (2)	0.0127 (19)	−0.0016 (19)
C2	0.066 (4)	0.046 (3)	0.059 (3)	0.022 (3)	0.024 (3)	0.005 (3)
C3	0.097 (5)	0.035 (3)	0.089 (5)	0.016 (3)	0.048 (4)	0.016 (3)
C4	0.091 (5)	0.037 (4)	0.102 (5)	−0.004 (4)	0.048 (4)	−0.009 (4)
C5	0.065 (4)	0.042 (3)	0.057 (3)	−0.008 (3)	0.022 (3)	−0.017 (3)
C6	0.055 (3)	0.035 (3)	0.044 (3)	0.002 (3)	0.021 (3)	−0.009 (2)
C7	0.057 (3)	0.041 (4)	0.028 (2)	−0.004 (2)	0.0160 (19)	−0.010 (2)
C8	0.073 (3)	0.044 (3)	0.057 (3)	−0.002 (3)	0.039 (3)	−0.004 (2)
C9	0.038 (3)	0.043 (3)	0.057 (3)	−0.006 (2)	0.021 (2)	−0.008 (3)
C10	0.037 (2)	0.045 (4)	0.060 (3)	0.003 (2)	0.018 (2)	0.002 (3)
C11	0.036 (3)	0.044 (3)	0.037 (3)	−0.010 (2)	0.011 (2)	−0.006 (2)
C12	0.052 (3)	0.039 (3)	0.054 (3)	−0.015 (3)	0.019 (3)	0.001 (2)
C13	0.082 (4)	0.036 (3)	0.058 (3)	−0.010 (3)	0.025 (3)	0.002 (3)
C14	0.066 (4)	0.041 (3)	0.050 (3)	0.013 (3)	0.019 (3)	0.010 (3)
C15	0.047 (3)	0.036 (3)	0.036 (2)	0.004 (2)	0.018 (2)	−0.001 (2)
N16	0.035 (2)	0.030 (2)	0.0298 (18)	−0.0029 (18)	0.0128 (16)	−0.0057 (16)
C17	0.047 (3)	0.041 (3)	0.026 (2)	−0.005 (2)	0.004 (2)	−0.005 (2)
C18	0.031 (3)	0.049 (3)	0.025 (2)	−0.005 (2)	0.008 (2)	0.000 (2)
C19	0.043 (3)	0.043 (3)	0.040 (3)	0.003 (2)	0.012 (2)	0.003 (2)
C20	0.055 (3)	0.049 (3)	0.057 (3)	−0.005 (3)	0.027 (3)	−0.009 (3)
C21	0.047 (4)	0.073 (5)	0.047 (4)	−0.018 (4)	0.019 (3)	−0.019 (3)
C22	0.039 (3)	0.091 (5)	0.037 (3)	−0.003 (3)	0.003 (2)	−0.001 (3)
C23	0.041 (3)	0.057 (3)	0.043 (3)	0.008 (3)	0.012 (2)	0.006 (3)
C24	0.049 (3)	0.046 (3)	0.039 (3)	0.003 (3)	0.015 (3)	0.000 (3)

### Geometric parameters (Å, °)

**Table:**

Zn1—O2	1.940 (3)	C10—H10A	0.9700
Zn1—N1	2.077 (4)	C10—H10B	0.9700
Zn1—N16	2.101 (4)	C11—N16	1.501 (6)
Zn1—Cl1	2.2189 (13)	C11—C12	1.530 (7)
O1—C24	1.213 (6)	C11—H11A	0.9800
O2—C24	1.280 (7)	C12—C13	1.523 (7)
N1—C2	1.491 (6)	C12—H12A	0.9700
N1—C10	1.509 (6)	C12—H12B	0.9700
N1—C6	1.514 (6)	C13—C14	1.522 (7)
C2—C3	1.508 (8)	C13—H13A	0.9700
C2—H2B	0.9700	C13—H13B	0.9700
C2—H2C	0.9700	C14—C15	1.529 (7)
C3—C4	1.545 (9)	C14—H14A	0.9700
C3—H3B	0.9700	C14—H14B	0.9700
C3—H3C	0.9700	C15—N16	1.502 (6)
C4—C5	1.522 (9)	C15—H15A	0.9700
C4—H4B	0.9700	C15—H15B	0.9700
C4—H4C	0.9700	N16—C17	1.488 (5)
C5—C6	1.519 (7)	C17—H17A	0.9700
C5—H5A	0.9700	C17—H17B	0.9700
C5—H5B	0.9700	C18—C19	1.387 (7)
C6—C7	1.532 (7)	C18—C23	1.399 (6)
C6—H6C	0.9800	C18—C24	1.506 (8)
C7—C8	1.526 (7)	C19—C20	1.381 (7)
C7—C17	1.527 (6)	C19—H19A	0.9300
C7—H7B	0.9800	C20—C21	1.377 (8)
C8—C9	1.520 (7)	C20—H20A	0.9300
C8—H8C	0.9700	C21—C22	1.370 (9)
C8—H8D	0.9700	C21—H21C	0.9300
C9—C10	1.526 (7)	C22—C23	1.397 (8)
C9—C11	1.527 (7)	C22—H22C	0.9300
C9—H9A	0.9800	C23—H23C	0.9300
			
O2—Zn1—N1	114.94 (15)	C9—C10—H10B	108.7
O2—Zn1—N16	98.37 (14)	H10A—C10—H10B	107.6
N1—Zn1—N16	89.06 (14)	N16—C11—C9	110.5 (4)
O2—Zn1—Cl1	113.80 (12)	N16—C11—C12	113.0 (4)
N1—Zn1—Cl1	124.97 (12)	C9—C11—C12	112.6 (4)
N16—Zn1—Cl1	107.58 (10)	N16—C11—H11A	106.8
C24—O2—Zn1	118.0 (4)	C9—C11—H11A	106.8
C2—N1—C10	108.6 (4)	C12—C11—H11A	106.8
C2—N1—C6	108.9 (4)	C13—C12—C11	112.8 (4)
C10—N1—C6	109.5 (4)	C13—C12—H12A	109.0
C2—N1—Zn1	112.1 (3)	C11—C12—H12A	109.0
C10—N1—Zn1	106.0 (3)	C13—C12—H12B	109.0
C6—N1—Zn1	111.6 (3)	C11—C12—H12B	109.0
N1—C2—C3	112.0 (4)	H12A—C12—H12B	107.8
N1—C2—H2B	109.2	C14—C13—C12	109.7 (4)
C3—C2—H2B	109.2	C14—C13—H13A	109.7
N1—C2—H2C	109.2	C12—C13—H13A	109.7
C3—C2—H2C	109.2	C14—C13—H13B	109.7
H2B—C2—H2C	107.9	C12—C13—H13B	109.7
C2—C3—C4	111.2 (5)	H13A—C13—H13B	108.2
C2—C3—H3B	109.4	C13—C14—C15	109.8 (4)
C4—C3—H3B	109.4	C13—C14—H14A	109.7
C2—C3—H3C	109.4	C15—C14—H14A	109.7
C4—C3—H3C	109.4	C13—C14—H14B	109.7
H3B—C3—H3C	108.0	C15—C14—H14B	109.7
C5—C4—C3	109.1 (5)	H14A—C14—H14B	108.2
C5—C4—H4B	109.9	N16—C15—C14	114.2 (4)
C3—C4—H4B	109.9	N16—C15—H15A	108.7
C5—C4—H4C	109.9	C14—C15—H15A	108.7
C3—C4—H4C	109.9	N16—C15—H15B	108.7
H4B—C4—H4C	108.3	C14—C15—H15B	108.7
C6—C5—C4	112.3 (5)	H15A—C15—H15B	107.6
C6—C5—H5A	109.1	C17—N16—C11	112.8 (3)
C4—C5—H5A	109.1	C17—N16—C15	112.5 (3)
C6—C5—H5B	109.1	C11—N16—C15	110.4 (3)
C4—C5—H5B	109.1	C17—N16—Zn1	107.2 (3)
H5A—C5—H5B	107.9	C11—N16—Zn1	108.9 (3)
N1—C6—C5	110.0 (4)	C15—N16—Zn1	104.6 (3)
N1—C6—C7	111.0 (4)	N16—C17—C7	113.0 (4)
C5—C6—C7	115.2 (4)	N16—C17—H17A	109.0
N1—C6—H6C	106.7	C7—C17—H17A	109.0
C5—C6—H6C	106.7	N16—C17—H17B	109.0
C7—C6—H6C	106.7	C7—C17—H17B	109.0
C8—C7—C17	109.4 (4)	H17A—C17—H17B	107.8
C8—C7—C6	108.3 (4)	C19—C18—C23	119.1 (4)
C17—C7—C6	116.8 (4)	C19—C18—C24	121.4 (4)
C8—C7—H7B	107.3	C23—C18—C24	119.5 (5)
C17—C7—H7B	107.3	C20—C19—C18	120.9 (5)
C6—C7—H7B	107.3	C20—C19—H19A	119.5
C9—C8—C7	106.4 (4)	C18—C19—H19A	119.5
C9—C8—H8C	110.5	C21—C20—C19	120.2 (6)
C7—C8—H8C	110.5	C21—C20—H20A	119.9
C9—C8—H8D	110.5	C19—C20—H20A	119.9
C7—C8—H8D	110.5	C22—C21—C20	119.5 (5)
H8C—C8—H8D	108.6	C22—C21—H21C	120.3
C8—C9—C10	108.3 (4)	C20—C21—H21C	120.3
C8—C9—C11	110.4 (4)	C21—C22—C23	121.5 (5)
C10—C9—C11	115.3 (4)	C21—C22—H22C	119.3
C8—C9—H9A	107.5	C23—C22—H22C	119.3
C10—C9—H9A	107.5	C22—C23—C18	118.8 (5)
C11—C9—H9A	107.5	C22—C23—H23C	120.6
N1—C10—C9	114.2 (4)	C18—C23—H23C	120.6
N1—C10—H10A	108.7	O1—C24—O2	124.5 (6)
C9—C10—H10A	108.7	O1—C24—C18	122.2 (6)
N1—C10—H10B	108.7	O2—C24—C18	113.3 (5)
			
N1—Zn1—O2—C24	65.5 (4)	N16—C11—C12—C13	−52.9 (5)
N16—Zn1—O2—C24	158.3 (4)	C9—C11—C12—C13	−179.0 (4)
Cl1—Zn1—O2—C24	−88.2 (4)	C11—C12—C13—C14	54.9 (6)
O2—Zn1—N1—C2	−79.6 (3)	C12—C13—C14—C15	−55.7 (6)
N16—Zn1—N1—C2	−178.4 (3)	C13—C14—C15—N16	57.1 (5)
Cl1—Zn1—N1—C2	70.6 (3)	C9—C11—N16—C17	50.9 (5)
O2—Zn1—N1—C10	38.7 (3)	C12—C11—N16—C17	−76.3 (5)
N16—Zn1—N1—C10	−60.1 (3)	C9—C11—N16—C15	177.7 (4)
Cl1—Zn1—N1—C10	−171.0 (2)	C12—C11—N16—C15	50.5 (5)
O2—Zn1—N1—C6	157.9 (3)	C9—C11—N16—Zn1	−68.0 (4)
N16—Zn1—N1—C6	59.1 (3)	C12—C11—N16—Zn1	164.8 (3)
Cl1—Zn1—N1—C6	−51.9 (3)	C14—C15—N16—C17	73.2 (5)
C10—N1—C2—C3	179.2 (4)	C14—C15—N16—C11	−53.7 (5)
C6—N1—C2—C3	60.0 (5)	C14—C15—N16—Zn1	−170.7 (3)
Zn1—N1—C2—C3	−64.0 (5)	O2—Zn1—N16—C17	−174.8 (3)
N1—C2—C3—C4	−57.7 (6)	N1—Zn1—N16—C17	−59.8 (3)
C2—C3—C4—C5	53.0 (7)	Cl1—Zn1—N16—C17	66.8 (3)
C3—C4—C5—C6	−54.2 (7)	O2—Zn1—N16—C11	−52.6 (3)
C2—N1—C6—C5	−59.4 (5)	N1—Zn1—N16—C11	62.5 (3)
C10—N1—C6—C5	−178.0 (4)	Cl1—Zn1—N16—C11	−170.9 (2)
Zn1—N1—C6—C5	64.9 (4)	O2—Zn1—N16—C15	65.5 (3)
C2—N1—C6—C7	172.0 (4)	N1—Zn1—N16—C15	−179.4 (3)
C10—N1—C6—C7	53.3 (5)	Cl1—Zn1—N16—C15	−52.8 (3)
Zn1—N1—C6—C7	−63.7 (4)	C11—N16—C17—C7	−50.6 (5)
C4—C5—C6—N1	58.4 (6)	C15—N16—C17—C7	−176.3 (4)
C4—C5—C6—C7	−175.3 (4)	Zn1—N16—C17—C7	69.2 (4)
N1—C6—C7—C8	−62.6 (5)	C8—C7—C17—N16	56.5 (5)
C5—C6—C7—C8	171.5 (4)	C6—C7—C17—N16	−67.0 (5)
N1—C6—C7—C17	61.4 (5)	C23—C18—C19—C20	0.6 (7)
C5—C6—C7—C17	−64.4 (5)	C24—C18—C19—C20	−177.3 (5)
C17—C7—C8—C9	−61.8 (5)	C18—C19—C20—C21	−0.6 (8)
C6—C7—C8—C9	66.6 (5)	C19—C20—C21—C22	−0.3 (8)
C7—C8—C9—C10	−63.1 (5)	C20—C21—C22—C23	1.3 (9)
C7—C8—C9—C11	64.1 (5)	C21—C22—C23—C18	−1.4 (8)
C2—N1—C10—C9	−170.5 (4)	C19—C18—C23—C22	0.4 (7)
C6—N1—C10—C9	−51.7 (5)	C24—C18—C23—C22	178.3 (5)
Zn1—N1—C10—C9	68.8 (4)	Zn1—O2—C24—O1	−7.4 (9)
C8—C9—C10—N1	57.6 (5)	Zn1—O2—C24—C18	173.0 (3)
C11—C9—C10—N1	−66.7 (5)	C19—C18—C24—O1	161.3 (6)
C8—C9—C11—N16	−58.9 (5)	C23—C18—C24—O1	−16.6 (9)
C10—C9—C11—N16	64.3 (5)	C19—C18—C24—O2	−19.1 (7)
C8—C9—C11—C12	68.5 (5)	C23—C18—C24—O2	163.1 (5)
C10—C9—C11—C12	−168.3 (4)		
